# Prevalence and Risk Factors for Chronic Kidney Disease in Belize: A Population-based Survey

**DOI:** 10.1016/j.lana.2021.100013

**Published:** 2021-07-17

**Authors:** Jian-Jhang Lin, Francis Morey, Hon-Yen Wu, Ju-Yeh Yang, Yu-Sen Peng, Deysi Mendez, Michel Chebat

**Affiliations:** a International Cooperation and Development Fund (TaiwanICDF), Taipei City, Taiwan; b Ministry of Health and Wellness, Belmopan, Belize; c Department of Internal Medicine, Far Eastern Memorial Hospital, New Taipei City, Taiwan; d School of Medicine, College of Medicine, National Yang Ming Chiao Tung University, Taipei City, Taiwan; e Institute of Epidemiology and Preventive Medicine, College of Public Health, National Taiwan University, Taipei City, Taiwan; f Department of Internal Medicine, National Taiwan University Hospital and College of Medicine, Taipei City, Taiwan

**Keywords:** chronic kidney disease, health surveys, prevalence, risk factors, Belize, Central America

## Abstract

**Background:**

Health resources supporting dialysis and chronic kidney disease (CKD) patients are limited in Central America, and little information about the prevalence and risk factors for CKD in this region is available.

**Methods:**

The Survey of Risk Factors for Chronic Kidney Disease was a population-based cross-sectional study conducted throughout Belize in 2017. The study aimed to assess the prevalence and risk factors for CKD via structured questionnaires and clinical measurements in Belizeans aged 20-55 years. A two-stage stratified sampling technique was applied. CKD was defined as an estimated glomerular filtration rate < 60 mL/min/1·73 m^2^ or the presence of proteinuria.

**Findings:**

A total of 7,506 adults with a mean age of 34·6 years old completed the survey; 53·2% were women. The overall CKD prevalence was 13·7%. Women had a higher CKD prevalence than men (14·8% vs. 12·5%), and the overall awareness of CKD was low (3·7%). The prevalences of stage 1, 2, 3a, 3b, 4, and 5 CKD were 2·85%, 2·93%, 6·59%, 1·10%, 0·18%, and 0·06%, respectively. Older age, female sex, Mestizo/Hispanic ethnicity, diabetes, hypertension, hypercholesterolaemia, and obesity were identified as independent risk factors for CKD.

**Interpretation:**

The prevalence of CKD was 13·7% in Belizeans aged 20-55 years. The study confirms the high burden of CKD in Belize and provides important epidemiological information for Central America. Case management systems and surveillance programmes targeting high-risk populations are crucial for ameliorating the burden of CKD.

**Funding:**

Capacity Building Project for the Prevention and Control of Chronic Renal Failure in Belize.

**RESEARCH IN CONTEXT**

**Evidence before this study**

We searched PubMed for population-based studies reporting the prevalence of and risk factors for chronic kidney disease (CKD) in Central American countries between Jan 1, 1970, and Dec 31, 2020, without language restrictions, using the search string "chronic kidney disease AND (prevalence OR risk factors OR incidence) AND (Central America OR Latin America)". Previous studies have reported that the CKD prevalence was 12·8% in adult population and 18% in several agricultural communities in El Salvador, 12% in the transisthmian zone of Panama, and 5-13% in different Pacific regions of Nicaragua. Most studies have found that older age, male sex, hypertension, and agricultural work were risk factors for CKD, but the results were mostly limited by regional data rather than nationwide surveys. The national CKD prevalence and its associated burdens in Belize have not been assessed previously.

**Added value of this study**

This study is, to our knowledge, the largest nationwide survey of CKD prevalence among Central American countries. In this population-based study throughout Belize, the overall prevalence of CKD was 13·7% among adults aged 20-55 years. The overall awareness of CKD was low, and stage 3a CKD accounted for half of the CKD cases. The prevalence of CKD was higher among women and older participants. Those with Mestizo/Hispanic ethnicity and residents of northern Belize also had a higher prevalence of CKD. In this study, older age, female sex, Mestizo/Hispanic ethnicity, diabetes, hypertension, hypercholesterolaemia, and obesity were identified as independent risk factors for CKD. The Mestizo/Hispanic ethnicity account for the highest impact on the prevalence of CKD among Belizeans aged 20-55 years.

**Implication of all the available evidence**

Estimating the national prevalence of CKD and assessing the impact of risk factors are central to the design of health programmes related to kidney care, and the results of this survey offer important information for stakeholders of the Belizean healthcare system. This national survey confirms the high burden of CKD in Belize and provides important epidemiological information for the region of Central America. A comprehensive CKD surveillance programme targeting high-risk populations, including those with Mestizo/Hispanic ethnicity, older age, hypertension, hypercholesterolaemia, diabetes, and obesity, is crucial for the early detection of CKD and ameliorating the burden of dialysis in Belize.

Alt-text: Unlabelled box

## 1 Introduction

Chronic kidney disease (CKD) has prevalence of 8-16% worldwide and accounts for the 16th leading cause of years of life lost [1,2]. Diabetes and hypertension are the main causes of CKD in most developed and developing countries, and other risk factors such as glomerulonephritis, obesity, dyslipidaemia, infectious diseases, analgesic abuse, herbal medications, pesticides, and environmental pollution, also contribute to CKD in many developing countries [1,[3], [4], [5]]. An epidemic of CKD of unknown cause, also known as Mesoamerican nephropathy, has emerged in Central America over the past two decades and primarily affects young men in agricultural communities [6], [7], [8]. Health resources supporting dialysis and CKD are quite limited in Central America, and knowledge about the prevalence and risk factors for CKD is mostly limited by regional data rather than nationwide surveys [1,9,10]. Previous studies have reported that the CKD prevalence was 12·8% in adult population and 18% in agricultural communities in El Salvador [11,12], 12% in two provinces of Panama [13], and 5-13% in different Pacific regions of Nicaragua [14], [15], [16]. However, the national CKD prevalence and its associated burdens in Belize and other Central American countries require more comprehensive surveys.

Early diagnosis and intervention are essential to reduce CKD-associated comorbidities, such as the need for dialysis and cardiovascular diseases [3,4,17,18]. Belize has the highest prevalence of diabetes and obesity in Central America [19,20], and it is important for the healthcare system to understand the actual burden of CKD throughout Belize. In this study, we aimed to estimate the CKD prevalence and identify the major CKD risk factors among adults aged 20-55 years in Belize.

## 2 Methods

### 2.1 Study participants

**Figure 1:**
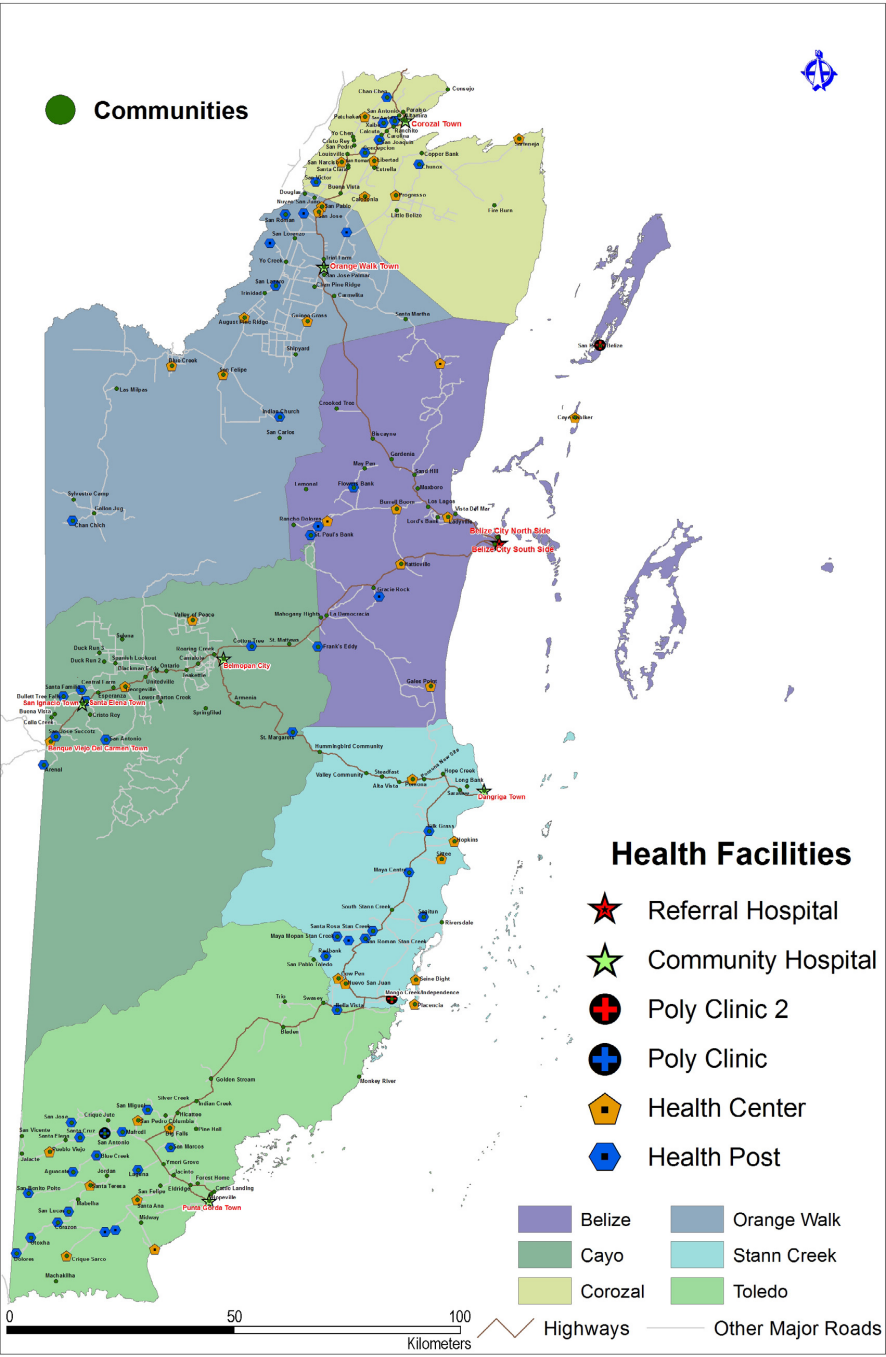
Administrative districts and public health facilities in Belize. The six administrative districts in Belize are shown in different background colours. The cluster sampling and survey were conducted in the indicated communities (green circles).

The Survey of Risk Factors for Chronic Kidney Disease (SRFCKD) was a population-based cross-sectional survey conducted throughout Belize in 2017 (Figure 1). The SRFCKD assessed the prevalence and distribution of and risk factors for CKD via structured questionnaires, body measurements, and blood and urine examinations in Belizeans aged 20-55 years. Citizens aged 20-55 years contributed to 45% of the national population and accounted for most of the working age population in Belize [21]. The sampling frame of the SRFCKD was constructed based on the most recent population and housing census by the Statistical Institute of Belize (SIB) in 2010, which divided the country into 527 enumeration areas (EAs) and each EA contained approximately 150 to 200 households [21].

A two-stage stratified sampling technique was applied to determine the sample population in the SRFCKD. The six administrative districts in Belize were treated as independent domains, and each domain was stratified into urban and rural areas. In the first stage of the sampling process, each district was allocated a certain number of EAs based on the number of households in the districts; these EAs were the primary sampling units. A total of 417 EAs were allocated in the first stage, with 195 in the urban and 222 in the rural areas. In the second stage of the sampling process, 24 households were selected via systematic random sampling from each EA allocated in the first stage. Among the sampled households, citizens aged 20-55 years old were eligible for recruitment. Individuals were excluded if they had a fever of up to 100·4°F or were hospitalized during the interview visits. Those who required maintenance dialysis, had received kidney transplantation, or refused to sign the informed consent form were also excluded. This study was approved by the Ethics Committee of the Ministry of Health and Wellness in Belize, and all participants provided written informed consent.

### 2.2 Questionnaire and clinical measurements

The SRFCKD study included structured questionnaires and clinical measurements, and all surveys were conducted in person by well-trained interview teams. The questionnaire consisted of household and individual components. The household component recorded information about all the members of the household and was used to identify eligible members for the individual component. The individual component collected personal information, including age, sex, ethnicity, region of residence, medical history, and lifestyle behaviours. The clinical measurements consisted of anthropometric measurements, blood pressure measurements, and laboratory blood and urine tests. Anthropometric measurements included body weight, body height, and waist circumference. Blood pressure was measured using an electronic sphygmomanometer (Digital Blood Pressure Monitor 7096, Dynarex Corporation, NY) after participants had sat for at least 15 minutes. Urine dipstick tests were used to detect protein and glucose. Random blood samples were drawn by phlebotomists in the field and delivered to laboratories of public facilities. Serum creatinine was measured using the Jaffe method, with calibration traceable to the isotope dilution mass spectrometry standard. All the blood samples were processed by trained medical staff, and all the laboratories used the same reagents (DiaSys Diagnostic Systems GmbH, Holzheim, Germany) and periodically calibrated analysers.

### 2.3 Participant characteristics

Participant data were recorded from the results of the questionnaires and clinical measurements. The estimated glomerular filtration rate (eGFR) was calculated using the 2009 Chronic Kidney Disease Epidemiology Collaboration (CKD-EPI) creatinine equation [22]. Proteinuria was defined as protein ≥ 1+ on the urine dipstick test [11,14]. According to age, participants were categorized into young (20-39 years) and middle-aged (40-55 years) groups [23]. To facilitate analysis among the various ethnic groups in Belize, we categorized participants into Mestizo/Hispanic, Black (Creole, Garifuna, and Black African), Maya (Ketchi, Mopan, and Yucatec), and other ethnicities. Participants who had completed at least Standard Five (7 years of primary education) were considered literate [21]. Body mass index (BMI) was calculated as body weight divided by body height squared (kg/m^2^). Obesity was defined as a BMI ≥ 30 kg/m^2^ [24]. Diabetes was defined by either a random blood glucose level ≥ 200 mg/dL or a self-reported medical history of diabetes. Hypertension was defined as the presence of any of the following: 1) a systolic blood pressure ≥ 140 mmHg, 2) a diastolic blood pressure ≥ 90 mmHg, or 3) a self-reported medical history of hypertension. Hypercholesterolaemia was defined as the presence of any of the following: 1) blood cholesterol ≥ 200 mg/dL, 2) blood low-density lipoprotein (LDL) ≥ 130 mg/dL, or 3) a self-reported medical history of hypercholesterolaemia. Gout was defined as a self-reported medical history of gout. Medical histories of nonsteroidal anti-inflammatory drug (NSAID) or herbal medicine use were obtained from the results of the individual questionnaires. Participants who reported themselves as current or former smokers were classified as those who had ever smoked. Frequent alcohol consumption was defined as consuming at least one alcoholic beverage per week [25]. For exercise habits, we categorized participants into those who exercised ≥ 90 minutes per week and those who exercised < 90 minutes per week [25,26].

### 2.4 Study outcomes

The study outcomes were the prevalence of CKD and the major risk factors for CKD in Belizeans aged 20-55 years. We defined CKD as the presence of any one of the following criteria: 1) an eGFR < 60 mL/min/1·73 m^2^ or 2) proteinuria [4,18]. CKD was further classified into stages 1-5 according to the eGFR [4,18]. Stages 1 and 2 were defined as eGFRs ≥ 90 mL/min/1·73 m^2^ and 60-89 mL/min/1·73 m^2^, respectively, with the presence of proteinuria [18]. Stages 3a, 3b, 4, and 5 were defined as eGFRs of 45-59, 30-44, 15–29, and < 15 mL/min/1·73 m^2^, respectively, regardless of proteinuria [18].

### 2.5 Statistical analysis

Statistical analyses were performed with SAS (version 9.4, SAS Institute, Cary, NC, USA), Stata (version 15, StataCorp LLC, College Station, TX, USA), and PASW Statistics for Windows (version 18.0, SPSS Inc., Chicago, IL, USA). The survey data were analysed while taking into account the complex survey design and weighted by the sampling weight. The sampling weight accounted for survey nonresponse, the population structure in the 2010 census (age, sex, and proportion of population in the sampling region), and the estimated size of Belize's population in 2017. The weighted data and results could be used to make inference to urban/rural population at the district and national level. Weighted values are presented as means (95% confidence interval [CI]) for continuous variables and as percentages (95% CI) for categorical variables. Differences in the distributions of continuous variables and categorical variables were examined using the independent two-sample *t*-test and chi-squared test, respectively. To examine the associations between potential risk factors and CKD (defined as either an eGFR < 60 mL/min/1·73 m^2^ or the presence of proteinuria), we first used univariate Poisson regression models to estimate the prevalence ratios (PRs), population attributable fractions (PAFs), and 95% CIs for the effects of each variable on CKD. Then, we used multivariate Poisson regression models with stepwise variable selection to identify the independent risk factors for CKD. We also used univariate and multivariate logistic regression models to evaluate the odds ratios (ORs) and 95% CIs of the potential risk factors for CKD. All the significant and nonsignificant variables were initially included in the multivariate regression model, and the final regression model was then established by excluding variables with a *P* value > 0·05 one at a time until all the remaining variables were significant. To assess the prevalence of CKD without classic risk factors, we further analysed the prevalence and characteristics of CKD patients without diabetes, hypertension, hypercholesterolaemia, obesity, and gout. A two-sided *P* value ≤ 0·05 was considered statistically significant.

### 2.6 Role of the funding source

The funders had no role in study design, data collection, data analysis, data interpretation, or writing of the report. All authors had full access to all the data in the study and had final responsibility for the decision to submit for publication.

## 3 Results

Of the 10,008 selected households, 7,883 (78·8%) completed household questionnaires, and 11,852 eligible adults were identified. A total of 10,343 (87·3%) adults completed the individual questionnaire, and 7,506 (63·3%) completed the clinical measurements.

### 3.1 Participant characteristics

Table 1 shows the weighted demographic and clinical characteristics of the 7,506 participants who completed the full questionnaire and clinical measurements. The weighted number of participants was 152,578, the mean age was 34·6 years, and 53·2% were women. More than half of the participants belonged to the Mestizo/Hispanic ethnic group (55·4%), followed by the Black (28·0%), Maya (10·7%), and other ethnic groups (6·0%). Most of the participants were literate (81·7%) and employed (66·9%). The Belize District (31·5%) and Cayo District (23·5%) accounted for the majority of the participants, and over half of the participants lived in rural areas (53·4%). The prevalences of diabetes, hypertension, hypercholesterolaemia, and obesity among the participants were 6·4%, 33·8%, 40·9%, and 40·9%, respectively. A total of 30·9% of the participants ever smoked, 16·1% consumed alcohol at least once a week, and 19·2% exercised at least 90 minutes per week. Furthermore, 47·0% of the participants ever used NSAIDs, and 50·2% ever used herbal medicines.

**Table 1:** Weighted demographic and clinical characteristics of the survey participants

Characteristics		All participants							Women							Men							*P* value§	
Unweighted No. of participants		7,506							4,482							3,024								
Weighted No. of participants		152,578							81,247							71,331								
(95% CI)	(	146,402		-	158,754		)	(	77,970		-	84,524		)	(	67,678		-	74,984		)			
Age, year		34·6	(	34·3	-	34·8	)		34·3	(	34·0	-	34·6	)		34·9	(	34·5	-	35·3	)		0·017	*
Age ≥ 40 years, %		32·4	(	31·2	-	33·5	)		30·9	(	29·5	-	32·3	)		34·0	(	32·3	-	35·7	)		0·002	*
Ethnicity, %																							0·202	
Maya		10·7	(	8·8	-	12·6	)		10·3	(	8·4	-	12·1	)		11·2	(	9·0	-	13·4	)			
Black		28·0	(	25·6	-	30·4	)		28·3	(	25·7	-	30·9	)		27·6	(	24·8	-	30·3	)			
Mestizo/Hispanic		55·4	(	52·5	-	58·2	)		55·9	(	52·9	-	58·8	)		54·8	(	51·5	-	58·1	)			
Others		6·0	(	4·5	-	7·4	)		5·5	(	4·1	-	6·9	)		6·5	(	4·8	-	8·2	)			
Literate, %		81·7	(	80·4	-	83·0	)		81·4	(	80·0	-	82·8	)		82·1	(	80·3	-	83·8	)		0·445	
Employed, %		66·9	(	65·6	-	68·2	)		47·2	(	45·3	-	49·1	)		89·3	(	88·1	-	90·6	)		<0·001	*
District, %																							0·121	
Corozal		12·7	(	11·7	-	13·6	)		12·8	(	11·8	-	13·8	)		12·5	(	11·2	-	13·9	)			
Orange Walk		13·2	(	12·2	-	14·2	)		13·3	(	12·2	-	14·4	)		13·1	(	11·9	-	14·3	)			
Belize		31·5	(	29·4	-	33·6	)		31·8	(	29·7	-	33·8	)		31·1	(	28·6	-	33·7	)			
Cayo		23·5	(	21·6	-	25·5	)		23·9	(	22·1	-	25·7	)		23·2	(	20·8	-	25·6	)			
Stann Creek		10·8	(	9·7	-	11·8	)		9·9	(	8·8	-	11·0	)		11·8	(	10·4	-	13·2	)			
Toledo		8·3	(	7·6	-	9·0	)		8·4	(	7·7	-	9·2	)		8·2	(	7·3	-	9·2	)			
Residence in rural area, %		53·4	(	51·4	-	55·5	)		52·4	(	50·4	-	54·4	)		54·6	(	52·0	-	57·2	)		0·048	*
Diabetes, %		6·4	(	5·8	-	7·1	)		7·2	(	6·4	-	8·0	)		5·6	(	4·7	-	6·5	)		0·009	*
Hypertension, %		33·8	(	32·4	-	35·1	)		31·9	(	30·3	-	33·6	)		35·9	(	33·7	-	38·0	)		0·003	*
Hypercholesterolaemia, %		40·9	(	39·4	-	42·5	)		41·3	(	39·5	-	43·0	)		40·5	(	38·3	-	42·7	)		0·538	
Obesity, %		40·9	(	39·6	-	42·3	)		49·4	(	47·6	-	51·3	)		31·2	(	29·2	-	33·3	)		<0·001	*
Gout, %		1·9	(	1·6	-	2·3	)		2·1	(	1·7	-	2·5	)		1·8	(	1·2	-	2·3	)		0·374	
Ever smoked, %		30·9	(	29·5	-	32·3	)		13·9	(	12·7	-	15·1	)		50·2	(	48·0	-	52·5	)		<0·001	*
Frequent alcohol consumption, %		16·1	(	14·7	-	17·4	)		8·3	(	7·2	-	9·5	)		24·8	(	22·7	-	27·0	)		<0·001	*
Ever used NSAIDs, %		47·0	(	45·4	-	48·6	)		50·1	(	48·3	-	52·0	)		43·4	(	41·2	-	45·5	)		<0·001	*
Ever used herbal medicines, %		50·2	(	48·5	-	51·9	)		51·4	(	49·5	-	53·3	)		48·9	(	46·5	-	51·3	)		0·051	
Exercised ≥ 90 min per week, %		19·2	(	18·0	-	20·4	)		16·9	(	15·5	-	18·3	)		21·7	(	19·9	-	23·6	)		<0·001	*

Note. Weighted values are presented as percentage (95% CI) for categorical variables and as mean (95% CI) for continuous variables. Variables are weighted using the sampling weights accounting for the population structure in the 2010 census and the estimated size of Belize's population in 2017. CI, confidence interval; NSAID, nonsteroidal anti-inflammatory drug.

§ *P* value for the test of difference between women and men.

⁎ *P* value ≤ 0·05.

Table 1 also compares the weighted clinical characteristics between women and men. Among the participants, women were younger and had a lower employment rate than men. Women had higher prevalences of diabetes and obesity but a lower prevalence of hypertension. In addition, women were more likely to use NSAIDs, and men were more likely to smoke, consume alcohol, and exercise. Supplementary Table 1 compares the characteristics between the 7,506 individuals who completed clinical measurements and the 2,837 who only completed questionnaire surveys. Among those who did not complete clinical measurements, there were a slightly younger age and lower percentages of female sex, illiteracy, unemployment, residence in rural area, and self-reported chronic diseases.

### 3.2 Prevalence of CKD and characteristics of the CKD patients

There was a total of 1,141 CKD patients, with a weighted number of 20,924 (95% CI 19,275-22,573) CKD patients. Table 2 shows the weighted prevalence of CKD among the participants. The overall CKD prevalence was 13·7%, and stage 3a CKD accounted for half of the CKD cases. The prevalences of stage 1, 2, 3a, 3b, 4, and 5 CKD were 2·85%, 2·93%, 6·59%, 1·10%, 0·18%, and 0·06%, respectively. Women (14·8%) had a higher CKD prevalence than men (12·5%) across stages of CKD. Only 3·7% of CKD patients reported having been told by a doctor that they had CKD, with the awareness of CKD being 3·7% among women and 3·6% among men. Only 2·8% of stage 1-3a CKD patients and 11·4% of stage 3b-5 CKD patients were aware of CKD.

**Table 2:** Weighted prevalence of CKD among the survey participants

	Unweighted No. of CKD patients	All participants (n=7,506)†						Women(n=4,482)†						Men(n=3,024)†						*P* value§	
All CKD	1,141	13·7	(	12·6	-	14·8	)	14·8	(	13·5	-	16·2	)	12·5	(	11·1	-	13·8	)	0·005	*
CKD stages																				0·017	*
Stage 1	171	2·85	(	2·20	-	3·50	)	3·07	(	2·25	-	3·89	)	2·60	(	1·80	-	3·41	)		
Stage 2	194	2·93	(	2·44	-	3·43	)	2·77	(	2·21	-	3·32	)	3·12	(	2·33	-	3·91	)		
Stage 3a	640	6·59	(	5·91	-	7·28	)	7·44	(	6·52	-	8·36	)	5·63	(	4·76	-	6·50	)		
Stage 3b	109	1·10	(	0·82	-	1·37	)	1·22	(	0·88	-	1·55	)	0·96	(	0·62	-	1·30	)		
Stage 4	21	0·18	(	0·10	-	0·27	)	0·25	(	0·11	-	0·39	)	0·11	(	0·01	-	0·21	)		
Stage 5	6	0·06	(	0·01	-	0·11	)	0·08	(	-0·01	-	0·17	)	0·03	(	-0·01	-	0·08	)		
Proteinuria	439	6·65	(	5·75	-	7·55	)	6·87	(	5·77	-	7·97	)	6·39	(	5·27	-	7·51	)	0·468	

Note. CKD, chronic kidney disease.

† Weighted prevalence (95% confidence interval) of CKD (%) using the sampling weights accounting for the population structure in the 2010 census and the estimated size of Belize's population in 2017.

§ *P* value for the test of difference between women and men.

⁎ *P* value ≤ 0·05.

Table 3 shows the weighted number and prevalence of CKD patients among the different subgroups. The prevalence of CKD was higher in participants aged ≥ 40 years (20·7%). Among the major ethnic groups, the Mestizo/Hispanic group had a higher prevalence of CKD (15·2%) than the Black (11·7%) and Maya (8·4%) groups. The prevalence of CKD was higher among participants who lived in the northern districts of Orange Walk (18·8%) and Corozal (16·6%) than among those who lived in the southern districts of Toledo (8·6%) and Stann Creek (9·5%). Participants who were illiterate (15·5%) had a higher prevalence of CKD than those who were literate (13·3%), but the prevalence of CKD was similar regarding the participants’ employment status and the residency in urban or rural areas. In addition, CKD was more prevalent among participants with diabetes (28·1%), hypertension (18·5%), hypercholesterolaemia (17·3%), obesity (16·4%), and gout (21·6%). Supplementary Table 2 compares the weighted characteristics between participants with CKD and those without CKD. CKD patients were older and had higher BMI, higher blood pressure, and larger waist circumference. Among the participants with CKD, there were higher percentages of female sex, Mestizo/Hispanic ethnicity, illiterate status, diabetes, hypertension, hypercholesterolaemia, obesity, and gout.

**Table 3:** Weighted number and prevalence of CKD patients within subgroups of survey participants

Subgroup	Unweighted No. of CKD patients	Weighted No. of CKD patients (95% CI)						Weighted prevalence of CKD (95% CI)						*P* value	
All CKD patients	1,141	20,924	(	19,275	-	22,573	)								
Age ≥ 40 years														<0·001	*
Yes	653	10,223	(	9,266	-	11,180	)	20·7	(	19·0	-	22·4	)		
No	488	10,701	(	9,251	-	12,153	)	10·4	(	9·1	-	11·7	)		
Sex														0·005	*
Women	725	12,043	(	10,817	-	13,269	)	14·8	(	13·5	-	16·2	)		
Men	416	8,881	(	7,836	-	9,928	)	12·5	(	11·1	-	13·8	)		
Ethnicity														<0·001	*
Maya	101	1,371	(	934	-	1,810	)	8·4	(	6·2	-	10·6	)		
Black	190	5,014	(	4,042	-	5,986	)	11·7	(	9·7	-	13·8	)		
Mestizo/Hispanic	736	12,877	(	11,440	-	14,314	)	15·2	(	13·8	-	16·7	)		
Others	114	1,662	(	1,070	-	2,254	)	18·2	(	14·1	-	22·3	)		
Literacy														0·036	*
Literate	876	16,585	(	14,938	-	18,232	)	13·3	(	12·1	-	14·5	)		
Illiterate	265	4,339	(	3,656	-	5,024	)	15·5	(	13·5	-	17·6	)		
Employment														0·939	
Employed	709	13,975	(	12,488	-	15,462	)	13·7	(	12·4	-	15·0	)		
Unemployed	432	6,949	(	6,092	-	7,806	)	13·8	(	12·3	-	15·3	)		
District														<0·001	*
Corozal	223	3,199	(	2,546	-	3,852	)	16·6	(	13·5	-	19·6	)		
Orange Walk	311	3,796	(	3,270	-	4,322	)	18·8	(	16·2	-	21·4	)		
Belize	185	6,237	(	5,076	-	7,398	)	13·0	(	10·7	-	15·2	)		
Cayo	224	5,038	(	3,922	-	6,154	)	14·0	(	11·5	-	16·5	)		
Stann Creek	97	1,556	(	1,098	-	2,014	)	9·5	(	6·8	-	12·2	)		
Toledo	101	1,098	(	790	-	1,406	)	8·6	(	6·4	-	10·9	)		
Residence area														0·976	
Rural	735	11,168	(	9,797	-	12,539	)	13·7	(	12·2	-	15·2	)		
Urban	406	9,756	(	8,445	-	11,067	)	13·7	(	12·0	-	15·4	)		
Diabetes														<0·001	*
Yes	168	2,754	(	2,320	-	3,188	)	28·1	(	23·8	-	32·3	)		
No	973	18,170	(	16,406	-	19,934	)	12·7	(	11·6	-	13·9	)		
Hypertension														<0·001	*
Yes	544	9,527	(	8,442	-	10,612	)	18·5	(	16·7	-	20·3	)		
No	597	11,397	(	10,082	-	12,714	)	11·3	(	10·0	-	12·5	)		
Hypercholesterolaemia														<0·001	*
Yes	601	10,804	(	9,548	-	12,060	)	17·3	(	15·6	-	19·0	)		
No	540	10,120	(	8,972	-	11,268	)	11·2	(	10·0	-	12·4	)		
Obesity														<0·001	*
Yes	601	10,238	(	9,152	-	11,324	)	16·4	(	14·8	-	18·0	)		
No	540	10,686	(	9,400	-	11,972	)	11·8	(	10·6	-	13·1	)		
Gout														0·003	*
Yes	45	639	(	456	-	821	)	21·6	(	15·6	-	27·6	)		
No	1,096	20,285	(	18,421	-	22,151	)	13·6	(	12·4	-	14·7	)		
Ever smoked															
Yes	298	6,246	(	5,334	-	7,158	)	13·3	(	11·5	-	15·1	)	0·520	
No	843	14,678	(	13,193	-	16,163	)	13·9	(	12·7	-	15·2	)		
Frequent alcohol consumption															
Yes	130	3,170	(	2,458	-	3,882	)	12·9	(	10·3	-	15·6	)	0·515	
No	1,011	17,754	(	16,099	-	19,409	)	13·9	(	12·7	-	15·0	)		
Ever used NSAIDs															
Yes	522	9,483	(	8,411	-	10,555	)	13·2	(	11·9	-	14·6	)	0·278	
No	619	11,441	(	10,153	-	12,731	)	14·1	(	12·7	-	15·6	)		
Ever used herbal medicines															
Yes	567	10,626	(	9,459	-	11,793	)	13·9	(	12·5	-	15·3	)	0·713	
No	574	10,298	(	9,106	-	11,492	)	13·6	(	12·1	-	15·0	)		
Exercised ≥ 90 min per week															
Yes	182	3,820	(	3,114	-	4,526	)	13·1	(	10·9	-	15·2	)	0·498	
No	959	17,104	(	15,461	-	18,747	)	13·9	(	12·7	-	15·1	)		

Note. Variables are weighted using the sampling weights accounting for the population structure in the 2010 census and the estimated size of Belize's population in 2017. CI, confidence interval; CKD, chronic kidney disease; NSAID, nonsteroidal anti-inflammatory drug.

⁎ *P* value ≤ 0·05.

Supplementary Table 3 shows that the weighted prevalence of CKD without classic risk factors was 2·65%, and women and men had a similar prevalence. About half of the CKD patients without classic risk factors were in the stage of 3, and the distribution across stages was similar to that in all CKD patients. Supplementary Table 4 compares the weighted characteristics between CKD patients with and without classic risk factors. CKD patients without classic risk factors were younger and had a lower percentage of female sex than CKD patients with class risk factors, whilst most of the other characteristics were similar among CKD patients with and without classic risk factors.

### 3.3 Risk factors for CKD

Table 4 shows the results of the univariate Poisson regression analyses for the weighted effects of various variables on CKD. Older age, female sex, and illiterate status were significantly associated with an increased risk of CKD. Compared with that in the Maya ethnic group, the risk of CKD was significantly increased in the Mestizo/Hispanic and Black ethnic group. Compared with those living in the southernmost district of Toledo, those living in other districts had a significantly higher risk of CKD. In addition, the risk of CKD was significantly increased in patients with diabetes, hypertension, hypercholesterolaemia, obesity, and gout. Table 5 shows the results of the multivariate Poisson regression analysis for the weighed effects of risk factors for CKD. We found that age ≥ 40 years (PR, 1·641; 95% CI, 1·597-1·686), female sex (PR, 1·182; 95% CI, 1·153-1·213), Mestizo/Hispanic ethnicity (PR, 1·716; 95% CI, 1·629-1·807), Black ethnicity (PR, 1·296; 95% CI, 1·226-1·371), diabetes (PR, 1·554; 95% CI, 1·500-1·610), hypertension (PR, 1·321; 95% CI, 1·287-1·357), hypercholesterolaemia (PR, 1·252; 95% CI, 1·220-1·284), and obesity (PR, 1·116; 95% CI, 1·087-1·145) were independent risk factors for CKD among Belizeans aged 20-55 years. Among the risk factors, the Mestizo/Hispanic ethnicity (PAF, 0·373; 95% CI, 0·343-0·402) accounted for the highest impact on the prevalence of CKD, followed by age ≥ 40 years (PAF, 0·191; 95% CI, 0·180-0·201), black ethnicity (PAF, 0·181; 95% CI, 0·144-0·217), hypertension (PAF, 0·111; 95% CI, 0·100-0·121), hypercholesterolaemia (PAF, 0·104; 95% CI, 0·092-0·116), female sex (PAF, 0·089; 95% CI, 0·075-0·102), obesity (PAF, 0·051; 95% CI, 0·039-0·063), and diabetes (PAF, 0·047; 95% CI, 0·043-0·051). The logistic regression analyses showed similar effects of the risk factors as that presented in the Poisson regression analyses (Supplementary Tables 5 and 6).

**Table 4:** Univariate Poisson regression model results for the weighted effect of each variable on CKD

Variable	PR	95% CI			*P* value		PAF	95% CI			*P* value	
Age ≥ 40 years	1·997	1·948	-	2·047	<0·001	*	0·244	0·238	-	0·250	<0·001	*
Women	1·190	1·160	-	1·221	<0·001	*	0·092	0·080	-	0·104	<0·001	*
Ethnicity												
Maya	Reference											
Black	1·395	1·317	-	1·476	<0·001	*	0·222	0·189	-	0·254	<0·001	*
Mestizo/Hispanic	1·811	1·717	-	1·909	<0·001	*	0·405	0·378	-	0·431	<0·001	*
Others	2·162	2·022	-	2·311	<0·001	*	0·294	0·277	-	0·311	<0·001	*
Literate	0·179	0·174	-	0·184	<0·001	*	-0·177	-0·183	-	-0·171	<0·001	*
Employed	0·995	0·969	-	1·022	0·714		-0·003	-0·021	-	0·014	0·714	
District												
Corozal	1·918	1·797	-	2·046	<0·001	*	0·356	0·331	-	0·381	<0·001	*
Orange Walk	2·179	2·045	-	2·322	<0·001	*	0·420	0·397	-	0·442	<0·001	*
Belize	1·503	1·414	-	1·598	<0·001	*	0·285	0·249	-	0·318	<0·001	*
Cayo	1·623	1·525	-	1·727	<0·001	*	0·315	0·283	-	0·346	<0·001	*
Stann Creek	1·095	1·017	-	1·178	0·016	*	0·051	0·010	-	0·089	0·014	*
Toledo	Reference											
Residence in rural area	0·998	0·973	-	1·023	0·846		-0·001	-0·015	-	0·012	0·846	
Diabetes	2·222	2·146	-	2·300	<0·001	*	0·073	0·071	-	0·075	<0·001	*
Hypertension	1·643	1·603	-	1·685	<0·001	*	0·179	0·172	-	0·185	<0·001	*
Hypercholesterolaemia	1·541	1·503	-	1·580	<0·001	*	0·181	0·173	-	0·190	<0·001	*
Obesity	1·397	1·362	-	1·432	<0·001	*	0·140	0·131	-	0·149	<0·001	*
Gout	1·593	1·486	-	1·709	<0·001	*	0·011	0·010	-	0·013	<0·001	*
Ever smoked	0·954	0·928	-	0·980	0·001	*	-0·015	-0·023	-	-0·006	0·001	*
Frequent alcohol consumption	0·933	0·901	-	0·967	<0·001	*	-0·011	-0·017	-	-0·005	<0·001	*
Ever used NSAIDs	0·936	0·912	-	0·960	<0·001	*	-0·031	-0·043	-	-0·019	<0·001	*
Ever used herbal medicines	1·024	0·999	-	1·051	0·060		0·012	-0·0004	-	0·025	0·058	
Exercised ≥ 90 minutes per week	0·941	0·911	-	0·972	<0·001	*	-0·011	-0·018	-	-0·005	<0·001	*

Note. Variables are weighted using the sampling weights accounting for the population structure in the 2010 census and the estimated size of Belize's population in 2017. CI, confidence interval; CKD, chronic kidney disease; NSAID, nonsteroidal anti-inflammatory drug; PAF, population attributable fraction; PR, prevalence ratio.

⁎ *P* value ≤ 0·05.

**Table 5:** Multivariate Poisson regression model for the weighted effects of variables on CKD

Variable	PR	95% CI			*P* value		PAF	95% CI			*P* value	
Age ≥ 40 years	1·641	1·597	-	1·686	<0·001	*	0·191	0·180	-	0·201	<0·001	*
Women	1·182	1·153	-	1·213	<0·001	*	0·089	0·075	-	0·102	<0·001	*
Ethnicity												
Maya	Reference											
Black	1·296	1·226	-	1·371	<0·001	*	0·181	0·144	-	0·217	<0·001	*
Mestizo/Hispanic	1·716	1·629	-	1·807	<0·001	*	0·373	0·343	-	0·402	<0·001	*
Others	2·065	1·935	-	2·203	<0·001	*	0·281	0·255	-	0·307	<0·001	*
Diabetes	1·554	1·500	-	1·610	<0·001	*	0·047	0·043	-	0·051	<0·001	*
Hypertension	1·321	1·287	-	1·357	<0·001	*	0·111	0·100	-	0·121	<0·001	*
Hypercholesterolaemia	1·252	1·220	-	1·284	<0·001	*	0·104	0·092	-	0·116	<0·001	*
Obesity	1·116	1·087	-	1·145	<0·001	*	0·051	0·039	-	0·063	<0·001	*

Note. Variables are weighted using the sampling weights accounting for the population structure in the 2010 census and the estimated size of Belize's population in 2017. CI, confidence interval; CKD, chronic kidney disease; PAF, population attributable fraction; PR, prevalence ratio.

⁎ *P* value ≤ 0·05.

## 4 Discussion

This is the largest nationwide survey of CKD prevalence among Central American countries. In this population-based survey conducted throughout Belize, there was an estimated number of 20,924 CKD patients which accounted for 13·7% of adults aged 20-55 years. The prevalences of stage 1, 2, 3a, 3b, 4, and 5 CKD were 2·85%, 2·93%, 6·59%, 1·10%, 0·18%, and 0·06%, respectively. Women had a higher CKD prevalence than men, and the overall awareness of CKD was low. The prevalence of CKD was higher in the Mestizo/Hispanic ethnic group, as well as residents of the northern districts of Orange Walk and Corozal. We identified that older age, female sex, Mestizo/Hispanic ethnicity, Black ethnicity, diabetes, hypertension, hypercholesterolaemia, and obesity were independent risk factors for CKD. In addition, the Mestizo/Hispanic ethnicity accounted for the highest impact on the prevalence of CKD among Belizeans aged 20-55 years.

The strengths of this study are the nationally representative sample and the large number of participants, accounting for 5% of Belize's population aged between 20-55 years. The survey data were analysed by taking into account the complex survey design and the population structure of the most recent census in Belize. This study was carefully designed with the assistance of statisticians from the SIB and conducted by trained field teams and medical staff. Therefore, we are confident that the prevalence of CKD in Belizeans aged 20-55 years was accurately estimated. Another strength of this study is that serum creatinine was measured using the same reagents with a calibration traceable to the isotope dilution mass spectrometry standard. We calculated eGFRs using the 2009 CKD-EPI creatinine equation, which is accurate throughout the full range of eGFRs [18,22]. This equation can be used for a wide range of ethnicities in the United States and Europe [27], and has been validated in the Mexican population [28]. As a result, the SRFCKD study was able to correctly categorize CKD from stage 1 to stage 5. Furthermore, the Mestizo/Hispanic ethnicity and older age showed a higher impact than chronic diseases, such as hypertension and diabetes, on the prevalence of CKD in population aged 20-55 years. The SRFCKD study confirms the high prevalence of CKD in Belize and provide valuable information regarding the priority for screening different high-risk populations. Estimating the national prevalence of CKD and assessing the impact of risk factors are central to the design of health programmes related to kidney care, and the results of this survey provide important information for stakeholders of the Belizean healthcare system.

The prevalence and awareness of CKD estimated in the SRFCKD study were similar to those in surveys among other low- and middle-income countries. Our analyses confirmed the association of CKD in Belize with classic risk factors, such as diabetes, hypertension, dyslipidaemia, and obesity [4]. In a cross-sectional study of 12 developing countries from six world regions, the CKD prevalence was 14·3% and the overall CKD awareness was 6%, with the awareness of only 8% in patients with stages 3b-5 CKD [29]. A cross-sectional survey of 4,817 adults in El Salvador reported a CKD prevalence of 12·8% in the general population, and stage 3 CKD contributed to over half of the CKD patients [12]. In a cross-sectional study of 2,388 adults from three agricultural communities in El Salvador, the prevalence of CKD was 18%, and the risk factors included older age, male sex, hypertension, agricultural work, and methyl parathion exposure [11]. Another cross-sectional study of 3,543 participants from the transisthmian zone of Panama reported a CKD prevalence of 12%, and the risk factors associated with CKD included older age, hypertension, previous myocardial infarction, and lower income [13]. In studies conducted in the Pacific regions of Nicaragua, the prevalence of stage 3-5 CKD ranged between 8-13% in northwestern Nicaragua, while it was only 5% in southwestern Nicaragua [[14], [15], [16],^30^]. Cross-sectional surveys in northwestern Nicaragua mostly showed that older age, male sex, hypertension, agricultural work, and dehydration were potential risk factors for CKD [15,16,30], while a cohort study in southwestern Nicaragua identified age, diabetes, hypertension, and sugarcane work to be associated with stage 3-5 CKD [14]. Unlike most previous studies, the present survey sampled participants across the whole country and enrolled a larger study population, allowing the analysis of nationally representative data with sufficient power.

The Mestizo/Hispanics, the major ethnic group in Belize, was identified as an independent risk factor for CKD in this study and was attributed to 37·3% of the CKD patients among Belizeans aged 20-55 years. A population-based study in the United States reported a CKD prevalence of 14·2% in Hispanic individuals, which was similar to that of 13·7% in non-Hispanic White individuals [31]. Despite a similar prevalence of CKD, the incidence rate of ESRD was 31·4% higher and the prevalence of ESRD was 55·3% higher in Hispanic than in non-Hispanic individuals [32,33]. The discrepancy suggests a more rapid progression of CKD in Hispanic individuals, which might be attributed to complex interplay among genetic susceptibility, socioeconomic factors, and classic risk factors such as diabetes, hypertension, and obesity [31,32]. This survey found a higher prevalence of CKD among participants living in the northern districts of Orange Walk and Corozal, of which about 80% of the population is composed of Mestizo/Hispanic individuals [21]. Moreover, sugarcane cultivation is a major component of the economy in Orange Walk and Corozal, and nearly half of the population in these northern districts engages in agricultural activities [21]. Recent reports have documented the emergence of Mesoamerican nephropathy in agricultural communities along the Pacific coast of Central America, involving those who work in sugarcane, cotton, corn, and banana fields [6,7]. This type of CKD is not caused by classic risk factors, such as diabetes or hypertension, and has been proposed to be due to multifactorial interactions among heat exposure, dehydration, toxins, agrochemicals, analgesics, and infections [[6], [7], [8],^10^]. This study found that 19% of the CKD patients did not have classic risk factors for CKD, however, it is not possible to explore the relationship between Mestizo/Hispanic ethnicity and agricultural work in this survey because we did not collect information about occupation type or work environment in detail. Further CKD health programmes should focus on the Mestizo/Hispanic ethnic group, and future health surveys in the agricultural regions of Belize should record the type of crops, labour conditions, heat exposure, use of pesticides, and hydrogeochemical features.

This study found a higher CKD prevalence in women than in men and identified female sex as an independent risk factor for CKD. Systematic reviews estimated a global CKD prevalence of 11·8% in women and 10·4% in men, and over two-thirds of population-based studies that assessed sex-specific prevalence reported a higher CKD prevalence in women [34,35]. However, the male to female ratio of CKD prevalence increases from stage 3 to stage 5 CKD, and the prevalence of end-stage renal disease (ESRD) in men surpasses that in women [36,37]. The higher prevalence of CKD in women could partially be explained by the longer life expectancy of women and might also be due to the potential underestimation of kidney function through the inappropriate use of eGFR equations [36]. On the other hand, the faster decline in kidney function in men might possibly be explained by unhealthier lifestyles in men or the protective effects of oestrogens in women [34,36]. Although women generally have a higher CKD prevalence than men in most geographical regions, variation still exists among Central American countries [36,37].

One limitation of the SRFCKD study is the cross-sectional design, which precludes the establishment of temporality between risk factors and the incidence of CKD. Because the diagnosis of CKD usually requires abnormalities of eGFR or kidney damage for at least 3 months [18], a single measurement of serum creatinine and urine protein might result some misclassification of the CKD status. However, most of the participants reported their usual health status and were unlikely to have experienced acute changes in kidney function, given that individuals with fever or hospitalization were excluded from the study. Second, individuals older than 55 years were not surveyed. Because CKD becomes more prevalent in the population with increasing age [23] and markers of kidney damage, such as structural changes in imaging studies or urine sediment abnormalities, were not assessed, the actual adult prevalence of CKD in Belize is expected to be higher than that estimated in this study and deserves more public health attention. Third, some diseases were characterized according to self-reported medical histories, which might have resulted in underestimation of potential risk factors. Because the blood samples were not collected in fasting status and the participants belonged to a relatively younger population, the prevalence of diabetes was lower and diabetes was attributed to cause only 5% of the CKD patients in the target population. Besides, the electronic sphygmomanometer used by the survey team had not been independently validated yet. However, our analyses still showed that diabetes, hypertension, and hypercholesterolaemia were independent risk factors for CKD, and the prevalences of most components of metabolic syndrome were similar to that in an earlier national survey [19,20]. Fourth, some participants provided local medication names for which ingredients were difficult to identify, and information bias could have influenced the effects of nephrotoxic medications in our analyses. This study found that a high percentage of participants with and without CKD used NSAIDs and herbal medicines; therefore, future studies on CKD risk factors in Belize should also focus on the medication habits and clarification of drug ingredients. Finally, about one quarter of the citizens who took the questionnaire surveys did not complete the clinical measurements, and the nonresponse rate was similar as other national survey for noncommunicable diseases among Central American countries [12,19]. As those who did not complete clinical measurements were slightly younger and had a lower prevalence of self-reported chronic diseases, study results might be influenced by nonresponse bias. Given the large sample size, this study still achieved adequate statistical power to identify the major risk factors for CKD. This national survey is an important first step in elucidating the actual burden and major risk factors for CKD in Belize, and additional longitudinal studies with a higher response rate are needed.

In conclusion, the CKD prevalence was 13·7% in Belizeans aged 20-55 years, and stage 3a CKD accounted for half of the CKD cases. The overall awareness of CKD was less than 4%, and the awareness was only about 10% among patients with stages 3b-5 CKD. This nationwide survey confirms the high burden of CKD in Belize and provides important epidemiological information for the region of Central America. A comprehensive CKD surveillance programme targeting high-risk populations, including those with Mestizo/Hispanic ethnicity, older age, hypertension, hypercholesterolaemia, diabetes, and obesity, is necessary for early detection and prevention of the progression of CKD. Implementing a CKD case management system and improving the healthcare infrastructure of renal replacement therapy were crucial for ameliorating the burden of CKD and dialysis in Belize.

## Declaration of interests

The authors declare no competing interests.

## Appendix Supplementary materials

Image, application 1
