# Supplementary material for: Prevalence and Risk Factors for Chronic Kidney Disease in Belize: A Population-based Survey
Source: Lancet Reg Health Am. 2021 Jul 17;1:100013. doi: 10.1016/j.lana.2021.100013 (PMC9903977; doi:10.1016/j.lana.2021.100013)
Supplement: Supplementary file 1 [file mmc1.pdf]

Supplementary Table 1. Demographic and clinical characteristics of the individuals who completed questionnaires

Characteristics	All individuals who completed questionnaires	With clinical measurements	Without clinical measurements	*P* value§
Unweighted No. of individuals	10,343	7,506	2,837	
Age, year	35·9 (10·1)	36·3 (10·2)	34·9 (9·8)	<0·001 *
Age ≥ 40 years	3,880 (37·5)	2,929 (39·0)	951 (33·5)	<0·001 *
Women	5,945 (57·5)	4,482 (59·7)	1,463 (51·6)	<0·001 *
Ethnicity				<0·001 *
Maya	1,235 (11·9)	1,043 (13·9)	192 (6·8)	
Black	2,357 (22·8)	1,578 (21·0)	779 (27·5)	
Mestizo/Hispanic	5,985 (57·9)	4,359 (58·1)	1,626 (57·3)	
Others	766 (7·4)	526 (7·0)	240 (8·5)	
Literate	8,306 (80·3)	5,926 (79·0)	2,380 (83·9)	<0·001 *
Employed	6,716 (64·9)	4,699 (62·6)	2,017 (71·1)	0·010 *
District				<0·001 *
Corozal	1,913 (18·5)	1,298 (17·3)	615 (21·7)	
Orange Walk	1,799 (17·4)	1,436 (19·1)	363 (12·8)	
Belize	2,159 (20·9)	1,334 (17·8)	825 (29·1)	
Cayo	2,155 (20·8)	1,462 (19·5)	693 (24·4)	
Stann Creek	1,082 (10·5)	950 (12·7)	132 (4·7)	
Toledo	1,235 (11·9)	1,026 (13·7)	209 (7·4)	
Residence in rural area	6,062 (58·6)	4,783 (63·7)	1,279 (45·1)	<0·001 *
Medical history of diabetes	571 (5·5)	464 (6·2)	107 (3·8)	<0·001 *
Medical history of hypertension	1,510 (14·6)	1,200 (16·0)	310 (10·9)	<0·001 *
Medical history of hypercholesterolaemia	1,142 (11·0)	910 (12·1)	232 (8·2)	<0·001 *
Medical history of gout	206 (2·0)	171 (2·3)	35 (1·2)	<0·001 *
Ever smoked	2,867 (27·8)	2,058 (27·4)	809 (28·6)	0·216
Frequent alcohol consumption	1,349 (13·0)	914 (12·2)	435 (15·3)	0·046 *
Ever used NSAIDs	4,752 (45·9)	3,593 (47·9)	1,159 (40·9)	0·984
Ever used herbal medicines	4,817 (46·6)	3,615 (48·2)	1,202 (42·5)	0·027 *
Exercised ≥ 90 min per week	1,966 (19·0)	1,350 (18·0)	616 (21·7)	0·002 *

Note. Values are unweighted, which are presented as the number (percentage) for categorical variables and as the mean (standard deviation) for continuous variables. NSAID, nonsteroidal anti-inflammatory drug.

§*P* value for the test of difference between individuals with and without clinical measurements.

**P* value ≤ 0·05.

Supplementary Table 2. Weighted demographic and clinical characteristics of the participants with and without CKD

Characteristics	With CKD	Without CKD	*P* value
Unweighted No. of participants	1,141	6,365	
Weighted No. of participants (95% CI)	20,924 (19,275 - 22,573)	131,654 (126,050 - 137,258)	
Age, year	38·8 (38·0 - 39·5)	33·9 (33·6 - 34·2)	<0·001 *
Age ≥ 40 years, %	48·9 (45·2 - 52·6)	29·7 (28·5 - 31·0)	<0·001 *
Women, %	57·6 (54·5 - 60·6)	52·6 (51·3 - 53·8)	0·005 *
Ethnicity, %			<0·001 *
Maya	6·6 (4·5 - 8·6)	11·3 (9·3 - 13·3)	
Black	24·0 (20·1 - 27·8)	28·6 (26·1 - 31·1)	
Mestizo/Hispanic	61·5 (57·0 - 66·1)	54·4 (51·5 - 57·3)	
Others	7·9 (4·9 - 11·0)	5·7 (4·4 - 7·0)	
Literate, %	79·3 (76·4 - 82·1)	82·1 (80·7 - 83·4)	0·036 *
Employed, %	66·8 (63·6 - 69·9)	66·9 (65·6 - 68·2)	0·939
District, %			<0·001 *
Corozal	15·3 (12·7 - 17·9)	12·2 (11·2 - 13·3)	
Orange Walk	18·1 (15·8 - 20·5)	12·4 (11·3 - 13·6)	
Belize	29·8 (26·1 - 33·5)	31·7 (29·5 - 34·0)	
Cayo	24·1 (20·1 - 28·1)	23·5 (21·5 - 25·4)	
Stann Creek	7·4 (5·7 - 9·2)	11·3 (10·2 - 12·5)	
Toledo	5·2 (3·8 - 6·7)	8·8 (8·1 - 9·6)	
Residence in rural area, %	53·4 (49·5 - 57·3)	53·4 (51·3 - 55·6)	0·976
Body mass index (kg/m^2)	30·7 (30·3 - 31·2)	29·2 (29·0 - 29·4)	<0·001 *
Systolic blood pressure (mmHg)	125·5 (123·9 - 127·0)	119·0 (118·4 - 119·7)	<0·001 *
Diastolic blood pressure (mmHg)	82·5 (81·3 - 83·6)	78·2 (77·8 - 78·6)	<0·001 *
Waist circumference (inch)	38·3 (37·9 - 38·8)	37·0 (36·8 - 37·2)	<0·001 *
Diabetes, %	13·3 (11·2 - 15·4)	5·4 (4·7 - 6·0)	<0·001 *
Hypertension, %	45·6 (42·1 - 49·1)	31·9 (30·5 - 33·3)	<0·001 *
Hypercholesterolaemia, %	51·6 (48·1 - 55·2)	39·2 (37·6 - 40·9)	<0·001 *
Obesity, %	49·2 (45·8 - 52·6)	39·6 (38·2 - 41·0)	<0·001 *
Gout, %	3·1 (2·1 - 4·0)	1·8 (1·4 - 2·1)	0·003 *
Ever smoked, %	29·9 (26·5 - 33·2)	31·0 (29·6 - 32·5)	0·529
Frequent alcohol consumption, %	15·2 (12·2 - 18·1)	16·2 (14·8 - 17·6)	0·515
Ever used NSAIDs, %	45·3 (42·0 - 48·7)	47·2 (45·6 - 48·9)	0·278
Ever used herbal medicines, %	50·8 (47·4 - 54·2)	50·1 (48·3 - 52·0)	0·702
Exercised ≥ 90 min per week, %	18·3 (15·3 - 21·2)	19·3 (18·1 - 20·6)	0·498

Note. Weighted values are presented as percentage (95% CI) for categorical variables and as mean (95% CI) for continuous variables. Variables are weighted using the sampling weights accounting for the population structure in the 2010 census and the estimated size of Belize's population in 2017. CI, confidence interval; CKD, chronic kidney disease; NSAID, nonsteroidal anti-inflammatory drug.
*P value ≤ 0·05.

Supplementary Table 3. Weighted prevalence of CKD without classic risk factors among the survey participants

	Unweighted No. of patients	All participants (n=7,506)†	Women (n=4,482)†	Men (n=3,024)†	*P* value§
All CKD	194	2·65 (2·16 - 3·13)	2·46 (1·85 - 3·07)	2·86 (2·15 - 3·56)	0·385
CKD stages					0·513
Stage 1	42	0·83 (0·49 - 1·17)	0·80 (0·35 - 1·26)	0·86 (0·43 - 1·29)	
Stage 2	28	0·50 (0·30 - 0·70)	0·38 (0·17 - 0·59)	0·64 (0·28 - 1·00)	
Stage 3a	104	1·09 (0·83 - 1·35)	1·10 (0·75 - 1·44)	1·08 (0·73 - 1·43)	
Stage 3b	17	0·20 (0·09 - 0·32)	0·14 (0·04 - 0·24)	0·28 (0·07 - 0·48)	
Stage 4	3	0·02 (-0·003 - 0·05)	0·04 (-0·01 - 0·09)	0	
Stage 5	0	0	0	0	
Proteinuria	76	1·41 (1·00 - 1·81)	1·25 (0·73 - 1·77)	1·58 (1·00 - 2·17)	0·389

Note. CKD, chronic kidney disease.

†Weighted prevalence (95% confidence interval) using the sampling weights accounting for the population structure in the 2010 census and the estimated size of Belize’s population in 2017.

§*P* value for the test of difference between women and men.

Supplementary Table 4. Weighted demographic and clinical characteristics of CKD patients with and without classic risk factors

Characteristics	All CKD patients	Without classic risk factors	With classic risk factors	*P* value§
Unweighted No. of participants	1,141	194	947	
Weighted No. of participants (95% CI)	20,924 (19,275 - 22,573)	4,036 (3,606 - 4,466)	16,888 (15,540 - 18,236)	
Age, year	38·8 (38·0 - 39·5)	32·6 (31·1 - 34·2)	40·2 (39·4 - 41·0)	<0·001 *
Age ≥ 40 years, %	48·9 (45·2 - 52·6)	24·1 (17·6 - 30·6)	54·8 (50·8 - 58·8)	<0·001 *
Women, %	57·6 (54·5 - 60·6)	49·5 (41·1 - 58·0)	59·5 (56·2 - 62·8)	0·034 *
Ethnicity, %				0·399
Maya	6·6 (4·5 - 8·6)	5·5 (1·2 - 9·7)	6·8 (4·9 - 8·8)	
Black	24·0 (20·1 - 27·8)	27·2 (19·1 - 35·3)	23·2 (19·2 - 27·2)	
Mestizo/Hispanic	61·5 (57·0 - 66·1)	57·0 (47·8 - 66·2)	62·6 (58·0 - 67·3)	
Others	7·9 (4·9 - 11·0)	10·3 (4·7 - 15·9)	7·4 (4·6 - 10·1)	
Literate, %	79·3 (76·4 - 82·1)	81·8 (75·9 - 87·6)	78·7 (75·6 - 81·7)	0·362
Employed, %	66·8 (63·6 - 69·9)	68·3 (60·8 - 75·8)	66·4 (63·0 - 69·9)	0·662
District, %				0·151
Corozal	15·3 (12·7 - 17·9)	17·2 (13·3 - 21·2)	14·8 (12·2 - 17·5)	
Orange Walk	18·1 (15·8 - 20·5)	20·2 (16·6 - 23·7)	17·7 (15·3 - 20·0)	
Belize	29·8 (26·1 - 33·5)	34·4 (29·2 - 39·7)	28·7 (25·1 - 32·3)	
Cayo	24·1 (20·1 - 28·1)	19·5 (15·4 - 23·6)	25·2 (21·0 - 29·4)	
Stann Creek	7·4 (5·7 - 9·2)	3·4 (2·2 - 4·6)	8·4 (6·5 - 10·3)	
Toledo	5·2 (3·8 - 6·7)	5·3 (1·8 - 8·7)	5·2 (4·1 - 6·4)	
Residence in rural area, %	53·4 (49·5 - 57·3)	47·6 (42·3 - 52·9)	54·8 (50·8 - 58·7)	0·125
Diabetes, %	13·3 (11·2 - 15·4)	0	16·4 (13·9 - 19·0)	NA
Hypertension, %	45·6 (42·1 - 49·1)	0	56·4 (52·7 - 60·1)	NA
Hypercholesterolaemia, %	51·6 (48·1 - 55·2)	0	64·0 (60·5 - 67·5)	NA
Obesity, %	49·2 (45·8 - 52·6)	0	60·9 (57·3 - 64·5)	NA
Gout, %	3·1 (2·1 - 4·0)	0	3·8 (2·6 - 4·9)	NA
Ever smoked, %	29·9 (26·5 - 33·2)	32·6 (25·2 - 39·9)	29·2 (25·5 - 33·0)	0·416
Frequent alcohol consumption, %	15·2 (12·2 - 18·1)	16·0 (9·3 - 22·6)	15·0 (11·9 - 18·0)	0·770
Ever used NSAIDs, %	45·3 (42·0 - 48·7)	42·0 (34·0 - 49·9)	46·1 (42·5 - 49·7)	0·330
Ever used herbal medicines, %	50·8 (47·4 - 54·2)	45·9 (38·5 - 53·2)	52·0 (48·3 - 55·8)	0·137
Exercised ≥ 90 min per week, %	18·3 (15·3 - 21·2)	23·5 (16·9 - 30·2)	17·0 (14·0 - 20·0)	0·044 *

Note. Weighted values are presented as percentage (95% CI) for categorical variables and as mean (95% CI) for continuous variables. Variables are weighted using the sampling weights accounting for the population structure in the 2010 census and the estimated size of Belize's population in 2017. CI, confidence interval; CKD, chronic kidney disease; NA, not applicable; NSAID, nonsteroidal anti-inflammatory drug.

§*P* value for the test of difference between CKD patients with and without classic risk factors.

**P* value ≤ 0·05.

Supplementary Table 5. Univariate logistic regression model results for the weighted effect of each variable on chronic kidney disease

Variable	Odds ratio	95% confidence interval	*P* value
Age ≥ 40 years	2·257	1·929 - 2·642	<0·001 *
Women	1·224	1·063 - 1·408	0·005 *
Ethnicity			
Maya	Reference		
Black	1·448	1·017 - 2·061	0·040 *
Mestizo/Hispanic	1·957	1·454 - 2·634	<0·001 *
Others	2·421	1·626 - 3·603	<0·001 *
Literate	0·834	0·704 - 0·989	0·037 *
Employed	0·994	0·855 - 1·155	0·939
District			
Corozal	2·099	1·456 - 3·026	<0·001 *
Orange Walk	2·451	1·748 - 3·437	<0·001 *
Belize	1·578	1·109 - 2·244	0·011 *
Cayo	1·724	1·204 - 2·468	0·003 *
Stann Creek	1·104	0·718 - 1·698	0·650
Toledo	Reference		
Residence in rural area	0·997	0·824 - 1·206	0·976
Diabetes	2·699	2·157 - 3·377	<0·001 *
Hypertension	1·789	1·535 - 2·086	<0·001 *
Hypercholesterolaemia	1·654	1·425 - 1·919	<0·001 *
Obesity	1·475	1·281 - 1·698	<0·001 *
Gout	1·757	1·211 - 2·549	0·003 *
Ever smoked	0·947	0·797 - 1·124	0·530
Frequent alcohol consumption	0·924	0·726 - 1·174	0·515
Ever used NSAIDs	0·926	0·805 - 1·065	0·279
Ever used herbal medicines	1·028	0·891 - 1·187	0·702
Exercised ≥ 90 min per week	0·932	0·761 - 1·142	0·498

Note. Variables are weighted using the sampling weights accounting for the population structure in the 2010 census and the estimated size of Belize’s population in 2017. NSAID, nonsteroidal anti-inflammatory drug.

*P value ≤ 0·05.

Supplementary Table 6. Multivariate logistic regression model for the weighted effects of variables on chronic kidney disease

Variable	Odds ratio	95% confidence interval	*P* value
Age ≥ 40 years	1·823	1·546 - 2·149	<0·001 *
Women	1·234	1·062 - 1·433	0·006 *
Ethnicity			
Maya	Reference		
Black	1·316	0·911 - 1·901	0·143
Mestizo/Hispanic	1·863	1·374 - 2·526	<0·001 *
Others	2·372	1·560 - 3·607	<0·001 *
Diabetes	1·800	1·435 - 2·257	<0·001 *
Hypertension	1·377	1·172 - 1·618	<0·001 *
Hypercholesterolaemia	1·314	1·121 - 1·542	<0·001 *
Obesity	1·142	0·983 - 1·327	0·083

Note. Variables are weighted using the sampling weights accounting for the population structure in the 2010 census and the estimated size of Belize's population in 2017.

*P value ≤ 0·05.
